# Group differences in physician responses to handheld presentation of clinical evidence: a verbal protocol analysis

**DOI:** 10.1186/1472-6947-7-22

**Published:** 2007-07-26

**Authors:** Danielle M Lottridge, Mark Chignell, Romana Danicic-Mizdrak, Nada J Pavlovic, Andre Kushniruk, Sharon E Straus

**Affiliations:** 1 Dept. of Mechanical & Industrial Engineering, University of Toronto, Toronto, Canada; 2 University Health Network, Toronto, Canada; 3 School of Health Information Science, University of Victoria, Victoria, Canada; 4 Dept. of Medicine, University of Calgary, Calgary, Canada

## Abstract

**Background:**

To identify individual differences in physicians' needs for the presentation of evidence resources and preferences for mobile devices.

**Methods:**

Within-groups analysis of responses to semi-structured interviews. Interviews consisted of using prototypes in response to task-based scenarios. The prototypes were implemented on two different form factors: a tablet style PC and a pocketPC. Participants were from three user groups: general internists, family physicians and medicine residents, and from two different settings: urban and semi-urban. Verbal protocol analysis, which consists of coding utterances, was conducted on the transcripts of the testing sessions. Statistical relationships were investigated between staff physicians' and residents' background variables, self-reported experiences with the interfaces, and verbal code frequencies.

**Results:**

47 physicians were recruited from general internal medicine, family practice clinics and a residency training program. The mean age of participants was 42.6 years. Physician specialty had a greater effect on device and information-presentation preferences than gender, age, setting or previous technical experience. Family physicians preferred the screen size of the tablet computer and were less concerned about its portability. Residents liked the screen size of the tablet, but preferred the portability of the pocketPC. Internists liked the portability of the pocketPC, but saw less advantage to the large screen of the tablet computer (F[2,44] = 4.94, p = .012).

**Conclusion:**

Different types of physicians have different needs and preferences for evidence-based resources and handheld devices. This study shows how user testing can be incorporated into the process of design to inform group-based customization.

## Background

Physicians are common users of mobile computers in the health care environment [1]. Given this trend, it is useful to obtain information about users' needs and preferences regarding these devices and relevant clinical practice tools available for use on them. Fundamental work in human computer interaction [2] has found differences in the order of twenty to one in users' speed and accuracy in common computing tasks, that users' individual differences can predict these differences, and that interfaces can be modified to account for them. In the medical domain, there are critical consequences due to failure to meet user needs, which include unused systems [3-6], wasted time[7], inadequate care [7] and physician errors [8]. This study examines group differences in responses to evidence-based resources on a tablet and pocketPC to make inferences about physicians' use of evidence resources and preferences for mobile devices.

Inconsistent access and application of relevant evidence is a significant cause of adverse events: research evidence, generated at an exponential rate, is not readily available to clinicians; when it is available, it is infrequently applied in clinical practice leading to care gaps [9-14]. Moreover, clinicians are limited by their inability to afford more than a few seconds per patient to find and assimilate relevant evidence [15-17].

Providing access to high-quality evidence resources at the point of care is one way to meet these challenges. Sackett and Straus evaluated the impact of evidence at the point of care and found that use of an 'evidence cart' increased the extent to which evidence was sought and incorporated into patient care decisions [17]. Clinicians were found to use evidence resources if they were easily accessible [18]. Practicing evidence based medicine (EBM) as little as once per month was related to better quality of care [19,20]. Using developments in information technology that have occurred since the Sackett and Straus study [17], this project aims to provide easily accessible evidence resources at the point of care using mobile computers.

Our objective was to develop a wireless medical information system that would bring the latest evidence to frontline physicians via handheld devices. The present study examines user needs to inform system design. Given that these are complex interventions aimed at improving the quality of care, a rigorous, iterative process of design, development and evaluation must occur prior to the actual clinical trial. Complex interventions are comprised of multiple components including behaviors, and methods of organizing and implementing these behaviors. The UK Medical Research Council has suggested a framework for development and evaluation of such complex interventions that includes exploring relevant theory and models [21]. During the initial phase, relevant theory is explored to optimize the choice of intervention and to predict major confounders. In the next phase, the components of the intervention are developed and their relationship to potential outcomes explored. For complex interventions involving health informatics technologies, we believe an extensive and methodologically rigorous process of design and development must occur with inclusion of the targeted users.

eHealth initiatives that are developed without including the end-user may lead to implementation failure [3,4,6]. A system that does not meet the needs of the users may cause the user to, at a minimum, waste time and provide lower quality care, [7] or even make errors [8]. The assessment of user needs is a unique challenge because of widely varying users, systems and settings. Several design methodologies that assess user-role and contextual needs have been introduced for medical interfaces [22,23].

Many surveys have been developed that identify user preferences for various mobile software and devices [1,24-37]. A notable user-group difference was that residents were found to have more expectations regarding mobile devices than faculty [25]. Further, the same study used work-role constructs to explain differences in frequency of accessing clinical data, patterns of email, pager and computer use [25]. In contrast, another survey study found no difference in usage preferences between physicians from different sub-specialties and medical students [28]. A third study used focus groups to elicit preferences about mobile computers. They found that physicians could be categorized into non-users, niche users, routine users and power users based on patterns of preferences [37]. Groups differed in their computer use, what the usage replaces (i.e., no, some or all paper) and their characteristics (respectively: skeptical, busy, open, technophiles).

Cognitive engineering principles can complement surveys to assess and meet user needs. The 'think-aloud' method elicits user knowledge that is useful for development [38]. Incorporating such methods into the design cycle can improve systems. For example, one such method was used to create a new medical record system for pediatric oncologists and was found to lower cognitive load and increase satisfaction [39].

Inclusion of the targeted end-users is a goal of this project: the Evidence at the Point of Care project (EPoCare). EPoCare is comprised of human factors engineers, computer scientists and practicing physicians working together to iteratively design, develop and evaluate clinical practice tools for mobile devices. The multi-disciplinary team used insights from an investigation into the use of evidence during clinical rounds [17] and an assessment of clinician needs for evidence at the point of care [40,41] to adapt paper and online versions of the evidence resources for mobile devices. During Phase I of the project [42] an HTML-based prototype was built with search interface screens and evidence resources. Group differences in needs and preferences were observed: family physicians tended to prefer short key messages, in contrast to general internists and internal medicine residents who wanted more detailed information [42]. Based on the Phase I findings, we decided to examine group differences more closely in Phase II to ensure that users' needs for these practice tools were met. A tablet and pocketPC were identified as suitable platforms for study because of comparable capabilities of concern to physicians (e.g., wireless capabilities, processing speed, battery life [43]) and also because of the fundamental differences that enabled us to examine the portability versus screen size tradeoff. Finding differences between groups of individuals would suggest opportunities and necessities for personalizing the presentation of clinical evidence according to the individual using that evidence. In summary, this research investigates the differences that impact physicians' needs for the content and presentation of clinical evidence on mobile devices, and the display formats and device form-factors that meet distinct groups' needs.

## Methods

This section first outlines the study, then describes the participants involved in the study, the session flow, the prototypes, the measures and the analysis that was carried out.

The methodology of a large-scale usability study was adopted in order to assess the differential customization requirements pertaining to identifiable subgroups of users [44]. Physicians from 3 user groups were identified in Phase I: general internists, family physicians, and internal medicine residents. General internists and family physicians were randomly selected from a sample of physicians who completed a survey on the use of mobile computers (n = 275 and n = 275, respectively)[45]. Staff physicians from university and non-university-affiliated settings were selected from Toronto, a large urban centre, and Sault St. Marie, a small urban centre. Internal medicine residents were recruited from the 120 residents in the General Internal Medicine Training Program at the University of Toronto. These physician groups were selected because they provided the bulk of care to patients in Ontario and are representative of the user groups for the proposed system.

After consent was obtained, 47 participants completed a 70-minute usability testing session that required them to complete a set of representative tasks using various evidence-based resources to answer relevant clinical questions. Participants were given two clinical scenarios to review that were relevant to their clinical practice. The scenarios were developed in consultation with a practicing family physician and a general internist. The family physician was asked to tape record his clinical questions during several clinics, while his workflow was observed by a human factors expert. The questions that arose during this physician's clinic were used to generate scenarios for the tasks. All unique patient identifiers were removed to preserve anonymity. Thus, one representative scenario for a specialized hospital setting was given to residents and general internists, one representative scenario for primary care clinic setting was given to general practitioners (shown below), and lastly one scenario appropriate for both the specialty hospital and primary care clinic environment was given to all participants. The first two scenarios were implemented on the pocketPC and the third on the tablet. Scenarios were designed to be equally difficult and representative. For example:

You see a 7-year-old child with asthma in your office. She is on fluticasone and salbutamol currently and was recently discharged from hospital following her 4th admission for asthma exacerbation. During the most recent admission, the dose of fluticasone was increased. Her mother is concerned about the impact of the additional dose of steroids on her daughter's growth. Together you formulate the question: In a child with asthma, do increased doses of inhaled corticosteroids lead to a decrease in growth?

Following a demonstration from the session facilitator, participants were asked to 'think aloud' as they used the prototype to search or browse for the answer to queries such as the one given above [46]. High-quality evidence resources were provided for use: Clinical Evidence (CE) [47] and Evidence-based Acute Medicine (EBOC) [48]. By high-quality evidence we mean that which has been appraised for validity and importance using methodologically explicit and rigorous techniques [49]. The content for each of these resources were provided to the research team in the form of XML files, which was formatted for the prototypes. Participants had access to both resources on both devices. Participants could search both resources, but could only browse one resource at a time.

**Figure 1:**
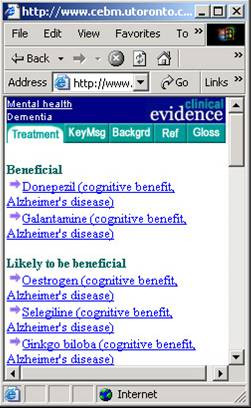
A screenshot from the pocketPC Implementation of the prototype showing CE Content.

**Figure 2:**
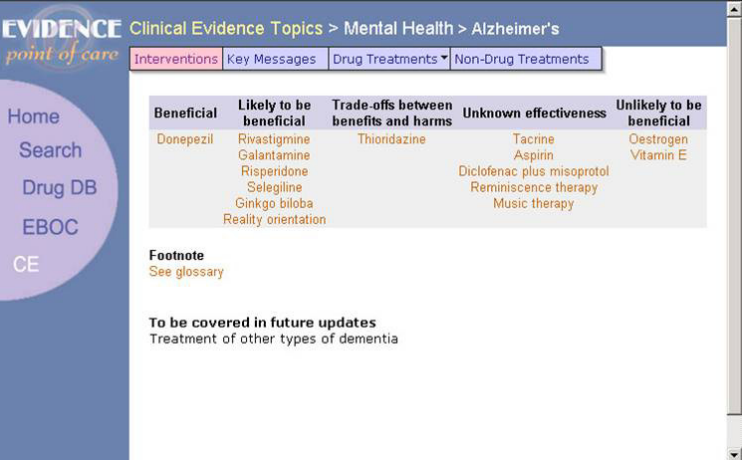
A screenshot from the tablet Implementation of the prototype showing CE Content.

HTML-based prototypes were developed for the tablet computer (screen: 4" × 6.4 ", resolution 640 × 480; device weight: approx. 1lb.; avg. battery life: 7 hours) and the pocketPC (screen: 2.26" × 3.02", resolution 240 × 320; device weight: 6.7ounces; avg. battery life: 7 hours). Both prototypes displayed the same clinical material, formatted differently to accommodate the different screen sizes and aspect ratios of the two devices (see Figure 1 for a screenshot of the pocketPC implementation and Figure 2 for a screenshot of the tablet implementation).

Each session was facilitated by a human factors engineering expert and observed by a research assistant. All sessions were videotaped and audiorecorded. The facilitator administered written questionnaires at the beginning and at the conclusion of the testing session. The initial questionnaire included background demographic questions, ownership and usage of technology and attitudinal questions about computers, based on the Technology Profile Inventory (Table 1) [50]. The participants were asked to respond to a series of statements at predetermined points in the session indicating their level of agreement (see Table 2). Responses were selected from a 5 point Likert-like scale anchored at 1 for "Strongly Agree" and 5 for "Strongly Disagree". Participants were instructed to verbalize the reasons for their choices. Other open-ended questions included those that clarified behaviors (for e.g., How did you decide what resource to pick (EBOC, CE)?), those that assessed user needs (for e.g., What do you think of the level of information presented?) and those that assessed user experience (for e.g., Would you be willing to carry around a device like the tablet or pocketPC during your work day to access this type of information?). The questions centered on the core usability attributes efficiency, effectiveness and user satisfaction, as per the ISO 9241 International Usability Standard. There was an additional focus on user preferences for devices and resources. An Exit Questionnaire focused on the user experience and context of future use. Some examples of the questions are: 'Would you be willing to carry around a tablet or pocketPC to access EBM information?', 'What (if any) other tasks would you use the device for?', and 'What (if any) additional tools would you like to have these devices?'. All sessions occurred in a research lab at the University Health Network in Toronto or in a clinical office at the Group Health Centre in Sault Ste Marie.

**Table 1:** True/False Items assessing 'Attitude towards computers'. Scores for items 1, 3, 5 are reversed, and scores for all items added.

I use computers only because they are necessary for work
I think that on-line shopping is a good idea
I don't want to know more about computers than I have to
Computers have a positive impact on my quality of life
I find dealing with computers to be frustrating
I am confident in my ability to master new skills with computers

**Table 2:** Likert scale items from the usability-testing session

1. The categories of the questions were useful.
2. The category that I should use for my question was clear.
3. I clearly understood what needed to be entered in each of the fields.
4. The description in the help files was useful.
5. This information would help me in the management of the patient in the scenario
6. I prefer seeing tables displayed in the text rather than having to tap on a link to see them.
7. It's easy to understand the table
8. I prefer the large window format rather than the small window with sub-windows.
9. This is the right amount of information on this drug
10. Online prescribing would be useful in my practice
11. The preset dosages are useful
12. This 'Limited Use Drug' (LUD) form would be useful in my practice.
13. I prefer the screen size of the tablet rather than that of the pocketPC.
14. I prefer the portability of the pocketPC rather than that of the tablet.

All audiotapes and videotapes were assigned a unique identifier and were transcribed verbatim. The 'think aloud' reports were analyzed using verbal protocol analysis [38]. A coding scheme developed during a preliminary phase of usability testing was modified and used for this analysis [42]. Coding categories included comprehensiveness of graphics and text, and problems in navigation and system functionality, among others (see Table 3). The measurement unit for coding was a statement or a self-contained utterance (see Table 4). Two research assistants independently coded a random sample of 15% of the transcripts in order to calculate inter-rater reliabilities. Frequency analyses were completed to assess how codes were distributed and to determine the frequency of both negative and positive comments. Chi-squared analyses were completed to determine differences between user groups. SPSS software was used to conduct one-way and two-way analyses of variance (ANOVA) to determine relationships between background variables and Likert-scale items. Repeated measures ANOVA was used to detect interactions between user groups, devices and coding frequencies.

**Table 3:** Coding Categories: Descriptions of Levels A through F

A. Specifies the main category of the code: usability, content or use/needs.
B. Identifies the portion of the prototype in which the point is being made about. An attribute to specify the location was optional. (E.g. Drug Database, Cascading Window).
C. Identifies the element on the screen. An attribute to specify the element was optional. (E.g. Format, Font).
D. Identifies the main point in subjects comment. (E.g. Usefulness)
E. Identifies the valence of the comment. (E.g. positive, negative, or neutral).
F. Identifies whether the comment was spontaneous or prompted. Any additional information was placed here.

**Table 4:** Examples of coded statements in a session transcripts

Example 1. The participant is describing a preference to have clickable drug names within the evidence-based resource that link to additional drug information.	
Participant	"you should be able to click on that, and it comes up with all the information, the dosing here, the, you know, side effects, and all that stuff, (...) [then] I would feel confident prescribing that drug...even though I have never prescribed it before (...)."
USE/NEED; CE; TEXT; USEFULNESS; NEUTRAL; SPONTANEOUS; "Drug names should link to more drug information."	
Example 2. The participant is answering a prompt from the investigator to explain why she finds the search input field categories useful.	
Participant	"why were the categories useful...consistent with evidence based medicine articles."
USABILITY; SEARCH; CATEGORIES; USEFULNESS; POSITIVE; PROMPTED	
Example 3. The participant is commenting on the Summary section in CE.	
Participant	"That's an awful lot of gibberish in the summary. Just a little hard. I tend to think in point form sometimes. I like the point forms the BMJ has taken on as to what these articles mean."
CONTENT; CE; LEVEL (of detail); NEGATIVE; SPONTANEOUS; "Wants summary in point form similar to BMJ".	

## Results

Forty-seven physicians participated in the study and their demographics are provided in Table 5. Compared to the national averages, our sample had a similar gender and age distribution, [51] but more Internet access (100% as compared to 72%). The mean age was 42.6 years and all residents were under 40 years of age. The age group composition of the general internists and family physician were similar: 1/5 of the groups was under 40 and 4/5, over 40. Approximately two thirds of physicians were in full-time clinical practice. Participants had varying degrees of computer expertise and attitudes towards computers. 91% of the sample reported that they would carry a device and 92.5% reported that they would use the evidence resource on it.

**Table 5:** Summary of Respondent Characteristics in the Sample

**Type**	**n**
family physician	17
general internist	17
medical resident	13
**Gender**	
male	31
female	16
**Age**	
under 30	8
30–39	12
40–49	13
50–59	11
60 and over	3
**Practice Setting**	
urban	37
semi-urban	10
**'Attitude Towards Computers' (Table 1)**	
1 (lowest)	4
2	5
3	7
4	6
5	13
6 (highest)	12
**Other**	
use e- medical databases	35
owned PDAs	27

Responses to the baseline questionnaire indicated a significant negative correlation between age and search engine use with use declining with increasing age (Pearson r = -.4, p < .01; Frequencies of usage were based on a monthly average: never, less than once, 1–5, 6–10, 11–15, more than 15). Usage of electronic databases also decreased with age (F[4,42] = 2.72, p = .042).

A total of 2367 events were extracted from the transcripts. The inter-rater reliability between the coders was good (k = .73).

Participants' verbalizations included responses to questions, prompts, and spontaneous thoughts. The ratio of spontaneous to prompted comments by participants was 55:45. Comments that focused on use or needs yielded more spontaneous thoughts (80:20, comparison across coding categories: Chi Square value > 18, df = 2, p < .001). Some comments in the use/needs included: "Statistics would be helpful because some issues are very individual and can't be answered by evidence" and "It would be useful if drugs were in a table, then we can have direct comparisons in specific areas".

The majority of comments about CE were concerned with the usability of the presentation of the content. A high proportion of the usability comments were positive: approximately 3:1, whereas the positive and negative comments were more equally split with respect to comments about content (comparison across coding categories: Chi Square value > 18, df = 2, p < .001). Participants commented that the depth and detail of the information was commendable, and that a greater variety of topics should be covered in future additions. Positive usability comments focused on navigability, scrolling and formatting issues such as colours and spacing. CE had a more positive response with a 30:70 ratio of negative to positive comments, whereas EBOC had an even ratio (comparison across coding categories: Chi Square value = 5.84, df = 1, p = .017). With respect to the content of CE, residents made fewer negative comments and more positive comments than family physicians and general internists (comparison across user groups: Chi Square value > 18, df = 2, p < .001). The verbal data indicated that family physicians tended to prefer the prominent bottom line presented at the beginning of each section within EBOC, while the other groups preferred the initial appearance of the evidence as presented in CE: "You get key messages and if you want to know more about it, then you click on it, and then if you want to know even more about that, you click on, so you go into more and more detail as you want...". The groups differed in their comments on the amount of detailed information: family physicians and residents had a high ratio of positive:negative comments (4:1) whereas the general internists' ratio was more even (comparison across user groups: Chi Square value = 12.06, df = 2, p = .002). Overall, residents had more positive comments and fewer negative comments than family physicians and internists (comparison across user groups: Chi Square value = 8.41, df = 2, p = .015).

When comparing usability comments for the browsing versus the searching function, browsing resulted in a higher positive to negative ratio than the search interface. There was an approximately 3:1 ratio of positive:negative comments for browsing, and a 1:1.5 ratio for searching (comparison of browse vs. search codes: Chi Square value = 12.02, df = 1, p < .001). While browsing, family physicians were more likely to take indirect routes (defined as extra pages visited outside of the direct path) in finding the answer to the clinical question. Residents also took more indirect routes than the general internists (comparison across user groups for categories 'direct', 'indirect', and 'not found'; Chi Square value = 11.76, df = 4, p = .019).

**Figure 3:**
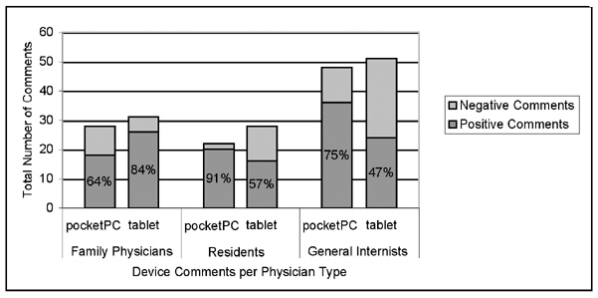
Ratio of Pos:Neg Comments about Devices from User Groups.

The pocketPC was better received than the tablet and had a significantly higher ratio of positive to negative comments than the tablet (comparison across devices: Chi Square value = 6.71, df = 1, p = .01). A significant interaction with the user groups and the ratio of positive to negative comments occurred between devices (F[2,44] = 4.94, p = .012). Residents and general internists had a near-even ratio of positive to negative comments for the tablet, while family physicians had a higher ratio of positive comments towards the tablet. Residents and internists had a higher ratio of positive to negative comments for the pocketPC than family physicians (See Figure 3). A qualitative investigation of the comments showed that family physicians' comments centered around the superiority of the tablet over their desktop PC. Residents and general internists focused on how the mobile nature of the pocketPC would support their workflow.

The user groups' device preferences stem from multiple factors. Residents made a larger proportion of comments about usability while family physicians commented more often on pragmatic, future uses (residents had a ratio of 4:1 of usability:use/needs comments, general internists, 2:1, family physicians, 1:1; comparison across user groups: Chi Square value = 7.65, df = 2, p = .02). For instance, when discussing the pocketPC, one resident suggested that a thinner, not smaller device, would be more convenient to carry. Representative statements from a family physician include the inclination to place the tablet on their office desk to replace their PC, and use it to print potential adverse effects from drugs for patients. Responses about device preferences (n = 294) included concerns related to portability (27%), display, or screen (27%), work setting (12%), tools (12%), data-entry (6%), and printing (3%). The user groups found that data-entry, display characteristics and work setting were of relatively equal importance to device selection. The user groups differed in their concerns on portability, printing and additional software or hardware tools. Family physicians were less concerned about portability and residents were more concerned about portability. Family physicians were concerned the most about printing functionality. Residents were very concerned about additional tools such as email, notes and calendar on their devices while general internists were less concerned about extra tools (Table 6; comparison across user groups for system features: Chi Square value = 11.13, df = 10, p = .004).

**Table 6:** Number of Coded Device-related Comments for each User-Group

*User Group*	*Data-Entry*	*Portability*	*Print*	*Display*	*Setting*	*Tools*
Family Physicians	6.8%	19.3%	8.0%	34.1%	18.2%	13.6%
Residents	5.5%	47.3%	0%	32.7%	9.1%	5.5%
General Internists	8.0%	32.1%	0.9%	27.7%	13.4%	17.9%

**Figure 4:**
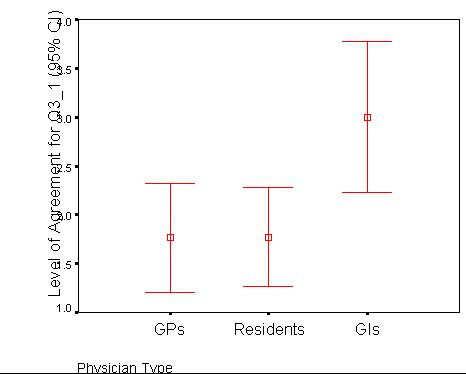
Mean Levels of Agreement for Q3_1 "I prefer the screen size of the tablet rather than that of the pocketPC." (1 = Strongly Agree, 5 = Strongly Disagree).

Additional data from the exit questionnaires[52] revealed that physicians had different preferences for the devices. Family physicians and residents preferred the screen size of the tablet (F[2,43] = 5.78, p = .006) (See Figure 4). Moreover, physicians who spent more time in the emergency department preferred the tablet computer (F[1,29] = 4.42, p < .05). Physicians who owned pocketPCs preferred the portability of the pocketPC more than those without previous experience (F[1,44] = 5.21, p = .027). In addition, physicians who used medical reference databases regularly preferred the portability of pocketPCs more than physicians who did not (F[1,24] = 4.54, p < .05). There were no significant differences between the urban and semi-urban groups.

## Discussion

This study investigated user needs for the presentation of clinical evidence on handheld computers. Physicians' work-role impacted form factor preferences. The evidence resources were found to be usable, though aspects of content such as details and statistics would benefit from customization. A summary of the main results is provided in Table 7.

**Table 7:** Summary of main user group differences

User groups	Content		Form Factor
	
	Resources	Sufficient Detail	pocketPC vs. tablet
Family Physicians	Family physicians preferred the bottom-line format of EBOC and wanted more focused answers from CE.	They liked the high level mode of the drill down format.	They wanted to use the device with a larger screen.
Residents	Residents' needs were met with CE as they responded with more positive comments.	The detailed mode for drill down was fine for residents.	Both residents and general internists liked the small-screen form factor.
General Internists	General Internists were positive and also critical of CE.	They were also critical of the amount of drill-down detail provided (they wanted more).	

The overall high frequency of positive usability comments about the Phase II prototype was encouraging. The high proportion of positive usability comments, versus the even proportion of content comments, demonstrates that although participants had mixed reviews about the evidence resources, participants found the prototype to be highly usable.

With regards to evidence resources, CE was better received than EBOC. Residents tended to have the most positive views, followed by family physicians and general internists. Family physicians seemed to prefer the bottom-line, or guideline-focused, format of EBOC, whereas the other groups tended to prefer the evidence presented in CE. General internists had more negative comments regarding the level of detail than residents and family physicians. It is possible that general internists within university-affiliated settings want more detail, while family physicians, who in our study were predominantly from non-university affiliated settings, want the clinical bottom-line. Since significant variability exists for these aspects of content, their format should be personalized and customized. In other words, the format of the evidence should be initially personalized to reflect the users' group needs. The users' work role would then determine the amount of evidence shown, the amount of detail provided about the evidence, and the placement of bottom-line guidelines. Additionally, users should be able to customize the interface by setting personal preferences. For example, a user may choose to show all, some or no tables of results, or to show only certain columns of tests that he or she is familiar with. Appropriate formatting settings to suit the user will insure that he or she is not overloaded with information extraneous to his or her interests. Extraneous information can distract users from information necessary for their clinical decision-making.

Browsing through the content was generally preferred over searching. This finding is likely due to the difficulty of inputting text on handheld devices through a touch-screen keyboard. Family physicians demonstrated greater difficulty in navigating to the correct medical answer than other groups. This effect may be due to a lesser familiarity with the evidence resources, as family physician tended to use medical databases (e.g., Medline, Harrison's, MD Consult) less often than the other groups [52].

The main result noted in this study was the difference in user groups' differing device preferences. Family physicians were more positive towards the tablet while residents and internists preferred the pocketPC. These findings are echoed in the exit questionnaire [52]. Family physicians preferred the screen size of the tablet and seemed less worried about its larger size. Family physicians' were more concerned about usefulness and less about usability. For example, they were interested in how the device would fit into their office setting and whether it could be used to print material for patients. Family physicians' preference for the tablet reflects that they tend to stay in their office, or move between adjacent rooms, when seeing patients. Residents liked the screen size of the tablet, but tended to prefer the portability of the pocketPC. The usability of the device and the types of tools available played a significant role in their preference for the pocketPC. Internists also preferred the portability of the pocketPC and saw less advantage in having the large screen of the tablet than family physicians and residents. Internists' choice likely stems from a greater need for portability due to the mobile nature of their work. In summary, portability seems to be less of a factor for family physicians, which suggests that a larger screen can be used to meet their needs. Portability is more of a factor for the internists, who valued increased mobility. The residents, however, wanted both the screen size of the tablet and the portability of the pocketPC. The younger residents seem to have higher expectations for technology and look forward to new devices on the market that are lighter and have more screen coverage on a smaller body [52]. It is worthwhile to note that the difference between devices is pronounced even though the tablet used in this study is smaller and lighter than those currently available on the market.

The physician factor was more sensitive to differences in user needs in terms of evidence resource format and device form-factor when compared to other potential predictors such as age and setting. However, members of physician groups tend to have some similar task and practice characteristics. Variables such as age, time spent practicing, and search engine use correlated with physician type in a cluster analysis [52]. The results of this study should be interpreted cautiously since other factors vary with physician type: for example, residents in this sample tended to be younger than the family physicians and general internists. Conversely, group differences reflect current population demographics and medical practice.

To date there are few studies that examine physicians' use of evidence-based resources on mobile devices 'in situ'. A small field study provided eleven residents with handhelds equipped with clinical evidence, an EBM calculator, a drug database and notes for a one month period [54]. Residents' reported that they liked the device and the information provided, however, they wanted more resources and found the wireless network unreliable. Findings from the present study confirm the pocketPC form-factor as appropriate for this user-group and could serve further to customize the EMB resources to increase likelihood of adoption. A recent study deployed smart phones to link 31 physicians to online medical resources for information retrieval during clinical and academic activities in a community hospital for a seven month period [55]. They found mixed reports regarding whether interns and residents located the target information and regarding the impact of the information, though participants reported high satisfaction. There were also usability concerns for the small screen and keyboard, which correspond with findings in this paper. User testing such as described in this study can serve to locate areas where the information presentation can be modified to better meet the users' expectations and needs.

A Cognitive Engineering approach to studying physician group needs is a valuable complementary method to surveys. Since surveys are self-reports, often removed from the situation under question, they may differ from actual clinical behavior [1,53]. Moreover, many reported surveys were not designed specifically for the purpose of detecting between-group differences. The current study is one of few (e.g.,[39]) that have carried out more in-depth task-based usability research employing multiple Cognitive Engineering assessment techniques. In addition, this study may be one of the first in this domain to quantify qualitative statements obtained from think aloud protocols in order to obtain a more reliable measure of their preferences. Finally, the study provides previously unreported description of user differences for mobile computers and evidence resources.

One of the limitations of this study is its sample size. A sample of 47 physicians is too small to confirm subgroup differences in the entire population, thus its conclusions serve to generate hypotheses for future research. Further, these devices were tested in a controlled laboratory study; investigation of usage in a clinical setting is essential to inform design prior to deployment. The information gained has been used to modify the prototypes according to individual clinicians' preferences to be further tested in subsequent clinical trials. The positive usability feedback suggests that the prototype has evolved to meet users' information needs. However, testing with a different sample of physicians who did not volunteer for this study is needed to confirm this finding. This study is a good example of how iterative usability testing can be used to drive interface design [56].

In accordance with clinicians' concern for additional tools, current directions of the EPoCare project include the design of electronic prescription and electronic health records for mobile devices to provide an integrated suite with the evidence resources. During this second phase of the EPoCare project, we examined hypotheses born out of the first phase of testing: how group differences interact with the usability of evidence resources on mobile devices. Applying the framework from the UK Medical Research Council [21] to the current study, we see that different form-factors may impact physicians' productivity and satisfaction. A future clinical trial will focus on these variables' relationship to quality of care. General internists, residents and family physicians should be included in any relevant clinical trials as they will likely experience different outcomes. Meanwhile, editors of evidence-based resources should consider personalizing the resources for different user groups in order to increase uptake and adoption. This work should also be extended to other user groups including nurses and patients. Finally, creators of eHealth tools for physicians and publishers of evidence resources should be aware that one size does not fit all. Targeted end users must be included in the design, development and testing of all of these innovations and their impact on clinical outcomes must be assessed.

## Conclusion

Previous research underlines the criticality of meeting user needs in medical informatics systems [6-8,22,23,39]. The present findings demonstrate that handheld presentation of clinical evidence should be personalized according to the requirements and preferences of different types of physicians. Regarding evidence resources, users demonstrate different needs for the amount of evidence shown and the level of detail provided. For example, only the conclusions from the strongest study designs should be shown to family physicians, versus the methods, statistical results, conclusions, and references from all studies for other groups. Family physicians prefer bottom-line guideline information more than the other groups. Regarding form-factor, family physicians prefer larger screens and are less concerned about mobility, while internists are most concerned about mobility. Residents present the most challenging design problem in their wish for both large screen size and high mobility. The strongest group differences were observed for physician type, with factors such as age, gender, and previous experience with the Internet and medical databases having relatively little effect on how the physicians responded to the prototype implementations. The information obtained in the current study demonstrates the value of adopting a rigorous framework of iterative development and evaluation concerning the use of mobile computers to improve clinical care.

## Competing interests

The author(s) declare that they have no competing interests.

## Authors' contributions

DML participated in the study design, carried out the study, performed some coding and data analysis, and drafted the manuscript. MC participated in the study design and performed statistical analysis. RDM participated in the study design, helped carry out the study and contributed to the interpretation of the data. NJP performed statistical analysis and contributed to the revision of the manuscript. AK participated in the conception and design of the study. SES conceived of the study, and participated in its design and coordination. All authors read and approved the final manuscript.

## Pre-publication history

The pre-publication history for this paper can be accessed here:


